# Pharmacy students’ perspectives on the initial implementation of a teaching electronic medical record: results from a mixed-methods assessment

**DOI:** 10.1186/s12909-020-02091-8

**Published:** 2020-06-09

**Authors:** Olga O. Vlashyn, Omolola A. Adeoye-Olatunde, Kimberly S. Illingworth Plake, Jamie L. Woodyard, Zachary A. Weber, Alissa L. Russ-Jara

**Affiliations:** 1 Purdue University College of Pharmacy, 575 W. Stadium Ave, West Lafayette, IN 47907 USA; 2 The Ohio State University Wexner Medical Center, 410 W 10th Ave, Columbus, OH 43210 USA

**Keywords:** Education, Pharmacy, Students, Electronic health records, Focus groups, Surveys and questionnaires

## Abstract

**Background:**

Electronic medical records (EMRs) have been used for nearly three decades. Pharmacists use EMRs on a daily basis, but EMRs have only recently been incorporated into pharmacy education. Some pharmacy programs have implemented teaching electronic medical records (tEMRs), but best practices for incorporating tEMRs into pharmacy education remain unknown. The objectives of this study were to 1) assess pharmacy students’ views and experiences with a tEMR; and 2) identify current learning activities and future priorities for tEMR use in pharmacy education.

**Methods:**

We used a mixed-methods approach, including three, two-hour student focus groups and a 42-item web-based survey to examine student perspectives of the tEMR. All first, second, and third year professional pharmacy students were eligible to participate in the survey and a focus group. Web-based survey items were measured on a 7-point Likert scale, and quantitative analyses included descriptive statistics. Two researchers independently coded transcripts using both deductive and inductive approaches to identify emergent themes. These analysts met and resolved any coding discrepancies via consensus.

**Results:**

Focus groups were conducted with 22 total students, with 6–8 students represented from each year of pharmacy training. The survey was completed by 156 students: 47 first year, 55 second year, and 54 third year. Overall, 48.7% of survey respondents altogether agreed or strongly agreed that using the tEMR enhanced their learning in pharmacy classes and laboratories. Qualitative data were organized into four major themes regarding tEMR adoption: current priorities for use within the pharmacy curriculum; tEMR benefits; tEMR barriers; and future priorities for tEMR use to prepare students for pharmacy practice.

**Conclusions:**

This study reveals pharmacy students’ perspectives and attitudes towards using a tEMR, the types of classroom activities that incorporate the tEMR, and students’ future suggestions to enhance the design or application of the tEMR for their learning. Our research findings may aid other pharmacy programs and promote more effective use of tEMRs in pharmacy education. In the long-term, this study may strengthen pharmacy education on EMRs and thus increase the efficacy and safety of pharmacists’ EMR use for patients’ medication management.

## Background

Along with physicians, nurses, and other healthcare providers, pharmacists utilize electronic medical records (EMRs) for tasks such as medication reconciliation, computerized physician order entry (CPOE), e-prescribing, and clinical decision support, but it is only recently that EMR training has been incorporated into pharmacy curricula [1]. Many healthcare disciplines, including nursing and medicine, are incorporating curricular content on EMR use and web-based patient centered modules to ensure trainees have adequate exposure to core medical content [2–6]. In the United States of America (USA), the Accreditation Council for Pharmacy Education (ACPE) recognizes professional communication and health informatics as two didactic content areas that are central to a contemporary, high-quality pharmacy education [7]. It is expected that, prior to becoming licensed, pharmacy students will explore and gain experience with technology-based communication tools and understand their impact on healthcare delivery, healthcare information, and patients’ medication management [7]. This expectation includes knowledge and proficiency with EMRs. In addition, the Joint Commission of Pharmacy Practitioners created a model of the Pharmacists’ Patient Care Process (PPCP), which recommends pharmacists use a patient-centered approach, in collaboration with other healthcare providers, to optimize patient care [8]. Pharmacists and pharmacy educators have begun describing and teaching this process within pharmacy curricula [9]. For several of the steps in the process, a teaching EMR (tEMR) could be used to enhance student learning.

Researchers have begun to examine the potential benefits of EMRs in pharmacy education. A 2015 survey by the American Association of Colleges of Pharmacy, a national organization representing pharmacy education in the USA, reported EMRs were most frequently used in the second and third professional pharmacy years to support teaching for clinical cases (77%) and pharmaceutical care lab courses (59%) [10]. A growing number of studies assess pharmacy students’ perceptions of EMRs, and these generally show students feel more confident and more prepared for advanced pharmacy practice experiences (APPEs) after using an EMR earlier in the pharmacy curriculum compared to being exposed to an EMR during their final year of pharmacy school [11–16]. Frenzel at a public university in the USA found that 94% of pharmacy students agreed that disease state management activities in an EMR were perceived to be beneficial in preparation for APPEs during the fourth year [11]. Results from Leibried et al. from a private university in the USA revealed that only 9.6% of students had prior experience with an EMR before being asked to use a simulated EMR in their first professional year; after the simulation, 72.6% of students felt more prepared to use an EMR in their upcoming hospital IPPE rotations [12]. Kirwin et al. demonstrated that despite 70% of pharmacy students at a private university in the USA in their third year of the program reporting prior experience with using EMRs, their comfort and confidence using EMRs was low. They conducted pre-post surveys and reported that students’ confidence significantly improved with the MEDITECH EMR [13].

We did not identify any studies that incorporated qualitative data (e.g., interviews, focus groups, etc.) to examine students’ EMR use in pharmacy curricula. Qualitative data can uncover themes that may not be easily captured in an online survey or a pre−/post-questionnaire. Furthermore, this type of data can provide deeper insights about students’ perceptions of new technologies since, unlike surveys, interviewers can probe further with individually tailored follow-up questions. Current studies on tEMR use in pharmacy education do not report using any adapted models or frameworks to guide information technology acceptance. Although it is known that EMRs impact the medication use process, best practices for incorporating EMRs into pharmacy practice and preparing students for future careers are currently unknown [17, 18]. Since pharmacy students are an integral part of the healthcare system and EMRs are ubiquitous in healthcare today, we conducted a study to gain a better understanding of pharmacy students’ early experience with a tEMR. The objectives of this study were to 1) assess pharmacy students’ views and experiences with a tEMR; and 2) identify current learning activities and future priorities for tEMR use in pharmacy education.

## Methods

### Study design and setting

We performed a mixed-methods, quantitative and qualitative study that consisted of a web-based survey and three focus groups with pharmacy students. This research was conducted with first, second, and third year pharmacy students at a large U.S. Midwestern College of Pharmacy in the USA. The College first implemented a tEMR (now known as the Regenstrief Institute EMR Clinical Learning Platform) for pharmacy students during the 2017–2018 academic year. The tEMR was available to all pharmacy instructors for use and was used in at least one class for all pharmacy cohorts during the year of our study (first year, second year, and third year students). The College’s primary objective for implementing the tEMR was for pharmacy students to gain exposure to an EMR during their didactic training, prior to pharmacy practice. The College did not specify tEMR learning objectives for pharmacy instructors’ use; the tEMR was freely available to all pharmacy instructors, who used the tEMR on an independent, voluntary basis, for any objectives or course activities they wished. This tEMR provides more than 10,000 de-identified patient records in a safe and secure learning environment. This platform allowed for learning from realistic clinical scenarios [19]. Besides the use of legitimate patient records, another unique feature of this tEMR is that it provides a communication feedback tool that allows instructors to provide feedback to individual students (J. Warvel, email communication, May 3, 2019). In this article, we present findings on student perspectives, but as part of the larger study, we also assessed pharmacy instructors’ perceptions of implementing a tEMR, including benefits and barriers, to further enhance the utilization of tEMRs within pharmacy school curricula. Study methods were approved by the University Institutional Review Board, and participants gave written informed consent.

### Study framework

This research was guided by the technology acceptance model (TAM) [20]. This model was developed in the 1980’s to assess technology acceptance and has been extensively used in healthcare [21–25]. The TAM framework has been applied to physicians, nurses, medical technicians, and pharmacists and the model has extensive application in explaining providers’ attitudes towards health information technology [26]. The original TAM suggests that an intention to accept technology is determined directly by individuals’ attitudes, perceived usefulness of the technology, and the perceived ease of use of the technology [27]. Perceived usefulness and perceived ease of use are of primary relevance for computer acceptance behaviors. Perceived usefulness is defined as an individual’s perception that the utilization of a particular technology will be advantageous in an organizational setting over a current practice. Perceived ease of use is the perception by an individual that the utilization of the new technology will be nearly painless or effortless [25]. It has since been expanded to include eight constructs: perceived usefulness; system quality; perceived self-efficacy; facilitating conditions; perceived ease of use; attitude toward using; behavior intention; and actual use [28]. We utilized the expanded TAM in our study as “prior research has found TAM as the most influential, commonly employed, and highly predictive model of information technology (IT) adoption.” [28]

### Web-based survey

#### Survey design

We conducted a 42-item, web-based survey (Qualtrics LLC, Provo, UT) to collect baseline data about students’ acceptance of the tEMR (Additional file 1 shows survey items). For this, 25 select items were adapted from a validated, 28-item survey based on the expanded TAM framework [28]. Seventeen questions were developed by the research team and added to obtain student demographics, to address pharmacy-specific questions, and capture data on tEMR usability. The survey included three sections of questions: students’ tEMR experience and use for pharmacy practice skills, students’ opinions regarding the tEMR, and students’ demographics and past experiences with EMRs in clinical care (e.g. EMR encountered outside the pharmacy curriculum, such as at a job or internship). These sections were comprised of 11, 25, and 6 questions, respectively. Items pertaining to students’ opinions of the tEMR were organized by the eight TAM constructs. These items were measured on a 7-point Likert scale where 1 = strongly disagree and 7 = strongly agree. The survey was piloted with two fourth year pharmacy students to assess questions for clarity and estimate the length of time needed for completion.

#### Survey participants

Students were eligible to participate if they were a first, second, or third year pharmacy student during the 2017–2018 academic year AND used or were asked to use the tEMR at least once for their coursework. The survey link was sent to all 454 pharmacy students through e-mail by a pharmacy administrator and remained open for completion for 1 month (April to May 2018). There were 151 first year, 156 second year, and 147 third year pharmacy students enrolled in the Doctor of Pharmacy program during the active survey period. If students chose to participate in the survey, they also had the chance to submit their name separate from their responses, to potentially receive one of three $30 gift cards per pharmacy year. Winners were selected at random and gift cards were e-mailed after the survey period closed.

#### Survey data analyses

SPSS software (IBM Corp, version 24, Armonk, NY) was used for statistical analysis. Survey responses received outside of the survey administration period and duplicate survey responses were excluded from statistical analyses. Descriptive statistics were performed. When appropriate, Chi-square or Kruskal-Wallis H tests were utilized to assess for statistically significant differences across the three pharmacy cohorts with respect to their demographics and survey responses. For items that stated “select all that apply”, a total count of choices was calculated. For example, we asked students to indicate the number of medication-related activities they use the tEMR for, and they could select “none” or choose up to eight different activities. These totals were summed across all three pharmacy cohorts and were used to gage students’ overall use of the tEMR. For all survey items, when item responses were significantly different between the pharmacy cohorts, we performed post-hoc pairwise tests to further assess potential differences between the pharmacy cohorts. An a priori alpha of ≤0.05 was considered significant for statistical analyses. Significance values were adjusted by Bonferroni correction for multiple comparisons [29].

### Focus groups

#### Focus group design

For student focus groups, we developed focus group questions (Additional file 2) and applied established methods to assemble and conduct three, 2-h focus groups. This included methods such as having a six to eight member homogenous group composition (i.e., grouped by year in program), preparing questions to help the moderator guide the group and ensuring that questions were open-ended, simple and conversational in nature [30]. The same two moderators conducted all three focus groups. One moderator had prior experience in qualitative methods, and the other moderator completed training on how to obtain a balanced input from a diverse group of students. Questions were developed to gather additional information on four key topics: students’ current use of the tEMR, benefits/perceived ease of use and usefulness, barriers/perceived ease of use and usefulness, and any ideas students had to improve the tEMR for student learning [31]. Sample questions included:

- What are the advantages of using the tEMR compared to the usual format for courses?
- Based on your experience, what are the strengths of the tEMR?
- Based on your experience, what are some barriers to your use of the tEMR?
- What are some EMR-related skills that you expect to need in pharmacy practice, but that have not yet been included in your coursework?
- If your use of the tEMR was optional for courses, would you still want to use it? Please explain.

A pilot focus group was conducted with a sample of 8 third year pharmacy students to assess the utility of the focus group guide and revise any ambiguous questions. (These students were excluded from the final data set for focus group analyses.)

#### Focus group participants

Students were eligible to participate if they were a first, second, or third year pharmacy student within the pharmacy curriculum during the 2017–2018 academic year, and they met screening criteria for the study. Participation in a focus group was voluntary, and we asked potential participants three screening questions in the sign-up link included in the e-mail:

1. Has any instructor ever asked you to use the teaching electronic medical record (tEMR)? (Yes/No)
2. Have you personally ever viewed OR entered information in the tEMR? (Yes/No)
3. Overall, what best describes your opinion of the tEMR? (Positive/Neutral/Negative)

The full census of eligible pharmacy students were contacted through e-mail by an administrative assistant and by class announcements by one of the research team members about participating in the study. For focus group recruitment, announcements were made by a student on the research team rather than an instructor to reduce the risk of students feeling coerced to participate. Furthermore, one of the focus group moderators had a student-student relationship with the 3rd year pharmacy student participants, so to manage potential conflicts, this focus group was led by the other moderator. If a student answered ‘No″ to *both* items 1 and 2, they were not eligible to participate in the focus group. Otherwise, students were eligible to participate, and we used these screening questions to purposely sample students with different opinions about the tEMR. Specifically, the third question was used to recruit a more diverse sample, with the goal of each focus group including at least 2 students that had a positive opinion, 2 students that had a negative opinion, and 2 students that felt neutral about the tEMR.

Focus groups were conducted in person in a private room and audio recorded. We projected the tEMR onto a large screen and asked participants to explain their comments by referring to the display. The references to the display were used to gather additional data to supplement audio recordings and handwritten notes. All students that answered at least one question during each of the three focus groups received a $30 gift card at the end of the session.

#### Focus group data analyses

Focus group audio recordings were transcribed by a third party (InfraWare, Inc., Terre Haute, IN). Transcripts were organized during analysis using MAXQDA 2018 (VERBI, v. 2018, Berlin, Germany) a qualitative data management software. An informatics researcher with expertise in qualitative methods guided the analysis process. Two research assistants independently read and coded the transcripts. A code book was developed and updated as appropriate throughout the coding process. Coding consisted of hybrid deductive/inductive approaches. First, interview transcripts were coded deductively into the four topic areas (see methods section, *Focus Group Design).* Then, data within each topic were analyzed inductively to identify emergent themes. The coders met to discuss line-by-line coding decisions. Coding disagreements were resolved through discussion [32]. Two analysts re-read the transcripts and codes for gaps, inconsistencies, and new interpretations to improve the validity of the analysis. The coded text was then grouped into major themes, and all three members of the analysis team met to agree upon final themes [33].

## Results

### Participants

#### Web-based survey

A total of 156 students (34.4% response rate) responded to the web-based survey, with 47 (31.1%), 55 (35.3%), and 54 (36.7%) first, second, and third year pharmacy students respectively. Students’ reported experience with real EMRs increased with year in pharmacy school (27.7%, 40.7%, and 94.0%, respectively). Altogether, 85 students (55.2%) reported past experience with an EMR outside of the curriculum, and the most commonly used EMRs were Epic (25.0%), Cerner (17.3%), McKesson (9.0%), and MEDITECH (8.3%). Students also reported using other EMRs, including CPSI EMR, Athena, Mayo Clinic’s EMR, VA/DoD system, and Sunrise Clinical Manager. Demographic characteristics were well balanced between respondents in all three pharmacy years (Table 1).

**Table 1:** Demographics of Pharmacy Students that Responded to the Survey

Characteristic	First year ***n*** = 47	Second year ***n*** = 55	Third year ***n*** = 54	Overall ***N*** = 156
Age in years, mean(SD)*	21.7 *(2.7)*^c^	23.5 (*4.9*)^d^	23.5 (*1.2*)	23.0 (*3.4*)^e^
Gender, n (%) female	31 (66.0)	42 (77.8)	42 (79.2)	115 (74.7)
Length of prior clinical EMR/EHR(s) exposure in a clinical setting*, n ^d,f^ (%)				152 (97.4)
None	26 (55.3)	30 (55.6)	2 (3.8)	58 (37.7)
Less than 3 months	8 (17.0)	8 (14.8)	27 (50.9)	43 (27.9)
4 to 6 months	6 (12.8)	2 (3.7)	8 (15.1)	16 (10.4)
7 months to 1 year	1 (2.1)	7 (13.0)	4 (7.5)	12 (7.8)
More than 1 year but less than 2 years	3 (6.4)	5 (9.3)	8 (15.1)	16 (10.4)
2 years or more	3 (6.4)	2 (3.7)	4 (7.5)	9 (5.8)
Experience using any type of EMR*, n (%)	13 (27.7)	22 (40.7)	50 (94.0)	85 (55.2)
Type of EMR used, n (%)				
Epic	6 (12.8)	5 (9.1)	28 (51.9)	39 (25.0)
Cerner	2 (4.2)	4 (7.3)	21 (38.2)	27 (17.3)
McKesson	4 (8.5)	7 (12.7)	11 (20.3)	22 (14.1)
MEDITECH	3 (6.4)	1 (1.8)	10 (18.2)	14 (9.0)
Other^a^	0	6 (10.9)	7 (13.0)	13 (8.3)
Intended practice type after graduation, n (%)				154 (98.7)
Industry	5 (10.6)	3 (5.6)	4 (7.5)	12 (7.8)
Ambulatory care	7 (14.9)	10 (18.5)	12 (22.6)	29 (18.8)
Hospital-based pharmacy	17 (36.2)	9 (16.7)	14 (26.4)	40 (26.0)
Community pharmacy	3 (6.4)	8 (14.8)	16 (30.2)	27 (17.5)
Independent pharmacy	0	2 (3.7)	1 (1.9)	3 (1.9)
Research	0	1 (1.9)	0	1 (0.6)
Unsure	14 (29.8)	16 (29.6)	5 (9.4)	35 (22.7)
Other^d^	1 (2.1)	5 (9.3)	1 (1.9)	7 (4.5)

* Significant difference between pharmacy class responses; *p* < 0.001

^a^Other EMRs used included CPSI, Athena, Mayo Clinic – Mick’s Last Word, VA/DOD system, Sunrise Clinical

Manager

^b^Other practice types included Academia, Long-term Care, Nuclear, Psychiatric, Veterinary

^c^For first-year students, *n* = 46 for this item

^d^For second-year students, *n* = 52 for this item

^e^For overall results, *n* = 151 for this item

^f^For overall results, *n* = 152 for this item

#### Focus groups

A total of 22 students participated in separate focus groups with 8, 8, and 6 students from the first, second, and third years respectively (Table 2). The average length of a focus group was 87 min.

**Table 2:** Student Focus Group Demographics

Characteristic	Participants, ***N*** = 22
Age in years mean (range)	22.3 (21–24)
Gender n (%) female	17 (77.3%)
Specific EMRs used in the past	
Epic	6 (27.3%)
Cerner	6 (27.3%)
Other	2 (9.1%)
None	8 (36.4%)
Overall, how comfortable are you with using the tEMR? (1 = very uncomfortable, 5 = very comfortable) Mean (range)	3.68 (2–4)

#### Variation in tEMR use and perspectives across pharmacy years in survey and focus groups

First year pharmacy students expressed needing more guidance and specifically requested adding a contact person on campus to help with tEMR needs. They also felt unsure about whether the tEMR used in the pharmacy curriculum was a true representation of what a real EMR is like in clinical practice. Second year pharmacy students felt that the tEMR was over-utilized in class. Students were conflicted between being given a progress note on paper that highlighted all the information that they would need to develop a care plan versus trying to find the pertinent information in the tEMR by themselves, as the latter could be time-consuming. In contrast, third year pharmacy students expressed interest in using the tEMR *more* often and had a very positive outlook overall. They mentioned several ideas for using the tEMR in pharmacy education, such as dosing medications, placing total parenteral nutrition (TPN) orders, and verifying orders. Based on focus group results, all pharmacy cohorts indicated the structure and organization of the tEMR was an area for improvement. Survey items where there were significant differences across pharmacy years are listed in Table 3.

**Table 3:** Survey items where there were significant differences across pharmacy years^a^

Survey Item	First year n (Mean Rank)	Second year (n, Mean Rank)	Third year (n, Mean Rank)	Chi-Square (***p***-value)	First year – Second year	Second year – Third year	First year – Third year
Overall, about how often did you use the tEMR during the 2017–2018 academic year?	47 (106.0)	55 (76.8)	53 (54.4)	40.974 (*p* < .01)	*p* < .01*	*p* = .01*	*p* < .01*
I have used the tEMR for the following medication-related activities.^b^	47 (76.3)	55 (98.9)	54 (59.6)	21.607 (*p* < .01)	*p* = .03*	*p* < .01*	*p* = .18
Using the tEMR better prepares me for my APPEs/P4 professional year.	47 (62.9)	54 (76.8)	53 (91.2)	10.803 (*p* = .02)	*p* = .32	*p* = .25	*p* < .01*
I have a generally favorable attitude toward using the tEMR for learning activities.	46 (71.9)	54 (68.2)	53 (90.4)	8.024 (*p* < .01)	*p* = 1.00	*p* = .02*	*p* = .10
What is your age in years?	46 (41.0)	52 (76.2)	53 (106.2)	57.303 (*p <* .01)	57.303 (*p* < .01)	*p* < .01*	*p* < .01*
What other EMR/EHR(s) have you been exposed to, if any? [Selected choice NONE vs. used at least 1 EHR.]	47 (56.6)	55 (67.6)	54 (108.7)	51.805 (*p* < .01)	*p* = .46	*p* < .01*	*p* < .01*
About how much exposure have you had from these EMRs in total (from previous question)?	47 (65.0)	54 (66.6)	53 (99.8)	21.938 (*p* < .01)	*p* = 1.00	*p* < .01*	*p* < .01*

Abbreviations: *APPE* advanced pharmacy practice experiences, *EHR* electronic health record, *EMR* electronic medical record, *P4* fourth year professional year, *tEMR* teaching electronic medical record

*Significant difference between pharmacy class responses, *p* < 0.05

^a^When appropriate, Chi-square or Kruskal-Wallis H tests were performed to determine any statistically significant difference across the three pharmacy classes’ demographics and survey responses. Total *N* = 156

^b^ “Select all that apply” question was transformed into a continuous variable with a minimum of 1 and maximum of 8

In terms of specific coursework, each of the three pharmacy year cohorts reported using the tEMR in pharmacy practice skills labs. Additionally, first year pharmacy students used the tEMR in the course ‘Pharmacy Case Studies,’ and second year pharmacy students used it during the course ‘Drug Information and Literature Evaluation.’ Third year pharmacy students did not report use beyond pharmacy practice skills lab. All pharmacy cohorts used the tEMR for creating Subjective, Objective, Assessment and Plan (SOAP) notes. In the sections below, additional survey and focus group results are organized according to the TAM constructs.

### Pharmacy practice skills

Survey respondents indicated the primary training students received for the tEMR was from pharmacy instructors (85.9%), watching online videos (77.6%), and completing an online quiz through the learning management system, Blackboard (69.9%) (Table 4). More than two-thirds (69.6%) of respondents agreed or somewhat agreed that the tEMR training they received was effective (Table 5). Over 80% of students used the tEMR for three activities: view a medication history, assess medication related problems, and review medication allergies (Table 4). Focus group results further highlighted that students were asked to collect patient information, prepare a SOAP note, perform a medication reconciliation, or interpret laboratory values (Additional file 3).

**Table 4:** Student survey responses related to use of the tEMR in pharmacy education

Survey Item	Response Options	n (%)
What training, if any, did you receive for the teaching electronic medical record (tEMR)? [Select all that apply]	Class instructor(s) provided guidance	134 (85.9)
	Completed online quiz on Blackboard	109 (69.9)
	Watched online videos	121 (77.6)
	tEMR BINGO	30 (19.2)
	None	1 (0.6)
	Other^a^	3 (1.9)
What best describes your experience using the tEMR during the 2017–2018 academic year? (*n* = 156)	I was asked to use the tEMR, but NEVER actually used it.	2 (1.3)
	I directly viewed/used but did not directly enter information in the tEMR.	136 (87.2)
	I directly viewed AND entered information in tEMR.	18 (11.5)
Overall, about how often did you use the tEMR during the 2017–2018 academic year?** (*n* = 155)	Daily	0
	Weekly	15 (9.6)
	Monthly	60 (38.7)
	A few times	80 (51.6)
	None	0
Overall, about how often did you use the tEMR during the 2017–2018 academic year?** (*n* = 155)	View medication history	154 (98.7)
	Assess medication related problems (MRPs)	126 (80.8)
	Review medication allergies	140 (89.7)
	Medication reconciliation	98 (62.8)
	Assess medication adherence	88 (56.4)
	Simulate communicating MRPs with health professional	68 (43.6)
	Assess cost-benefit, formulary, and/or epidemiology principles to medication-related decisions	13 (8.3)
	Verify medication orders	38 (24.4)
I have used the tEMR for the following medication-related activities:**^b^ [Select all that apply]	View medication history	77 (49.4)
	Assess medication related problems (MRPs)	74 (47.4)
	Review medication allergies	49 (31.4)
	Medication reconciliation	71 (45.5)
	Assess medication adherence	70 (44.9)
	Simulate communicating MRPs with health professional	57 (36.5)
	Assess cost-benefit, formulary, and/or epidemiology principles to medication-related decisions	70 (44.9)
	Verify medication orders	40 (25.6)
	The tEMR does NOT need to be improved for medication-related activities	15 (9.6)
I also used the tEMR for these other activities:^b^ [Select all that apply]	View a MEDICAL history	139 (89.1)
	Determine a patient’s health-related needs	104 (66.7)
	Interpret laboratory test results	141 (90.4)
	Assess problems NOT related to medications	68 (43.6)
	Simulate a handoff or transition of care	26 (16.7)
	Assess or document immunizations	19 (12.2)
	Write a note for patient documentation	46 (29.5)
	Document other patient-specific information	21 (13.5)
	I did NOT use the tEMR for any other activities	3 (1.9)
The tEMR needs to be improved to effectively support these other activities:^b^ [Select all that apply]	View a MEDICAL history	62 (39.7)
	Determine a patient’s health-related needs	53 (34.0)
	Interpret laboratory test results	46 (29.5)
	Assess problems NOT related to medications	54 (34.6)
	Simulate a handoff or transition of care	57 (36.5)
	Assess or document immunizations	56 (35.9)
	Write a note for patient documentation	58 (37.2)
	Document other patient-specific information	43 (27.6)
	The tEMR does NOT need to be improved for any other activities	23 (14.7)

^a^Other text included “lab and case studies” and “practicing with MDs’ cases in lab”

^b^Transformed into continuous variable by computing a total count of choices selected to determine any statistically significant difference between pharmacy class responses

** Significant difference between pharmacy class responses; p < 0.001

**Table 5:** Student Survey Responses Related to Use of tEMR in Pharmacy Education^a^

Domain/survey item	n	Rating Median (IQR)a	Strongly Disagree %	Disagree %	Somewhat Disagree %	Neither Agree/Disagree %	Somewhat Agree %	Agree %	Strongly Agree %
**Pharmacy Practice Skills**									
The tEMR training I received was effective.	154	**5 (2)**	1.3	5.8	14.2	3.9	36.1	33.5	4.5
The tEMR training (instructor guidance, online materials, etc.) was available to me at the time that I needed it.	156	**6 (1)**	1.9	3.9	5.8	5.2	19.4	51.6	12.3
**System Quality (SQ)**									
I am satisfied with the use of the tEMR system for student learning.	155	**5 (3)**	1.9	10.3	18.7	6.5	32.9	24.5	5.2
I am satisfied with the tEMR system for finding relevant patient information.	155	**5 (3)**	5.2	11.0	17.4	7.7	31.0	24.5	3.2
I am satisfied with the quality of the tEMR’s clinical content for my learning.	155	**5 (2)**	1.3	6.5	10.3	9.7	38.1	29.7	4.5
I am satisfied with the tEMR’s learning relatedfunctions (e.g., external resources, contact support, tEMR tutorials).	155	**5 (2)**	1.9	9.7	12.3	25.8	21.9	25.8	2.6
**Perceived Self-Efficacy (PSE)**									
Overall, I feel confident using the tEMR for my pharmacy classes and laboratories.	154	**5 (2)**	1.3	7.1	12.3	7.1	40.9	26.0	5.2
Overall, I feel confident using the tEMR to work on clinical scenarios.	155	**5 (2)**	1.9	8.4	12.3	8.4	41.3	24.5	3.2
After using the tEMR, overall, I feel more confident in my ability to use real electronic medical records (EMRs) as a pharmacist.	155	**5 (2)**	2.6	7.1	6.5	10.3	33.5	34.8	5.2
**Perceived Ease of Use (PEOU)**									
The on-screen instructions provided by the tEMR are clear.	155	**5 (3)**	3.2	9.7	17.4	10.3	34.8	22.6	1.9
Completing tasks with the tEMR requires minimal mental effort.	155	**3 (3)**	10.3	20.0	25.8	8.4	22.6	12.3	0.6
I found it easy to get the tEMR to do what I want it to do.	155	**3 (3)**	11.0	18.1	21.9	12.9	21.9	12.3	1.9
When working on activities, I found it easy to locate: patient(s)	155	**6 (1)**	0	2.6	0	1.3	9.7	49.7	36.8
When working on activities, I found it easy to locate: patient information	154	**5 (1)**	1.9	7.8	9.1	2.6	35.1	37.7	5.8
When working on activities, I found it easy to locate: pertinent lab values	155	**5 (3)**	0.6	12.3	14.2	7.7	30.3	34.2	0.6
When working on activities, I found it easy to locate: patient medication	155	**5 (3)**	3.2	15.5	17.4	7.1	30.3	23.2	3.2
**Facilitating conditions (FC)**									
It was easy to learn how to use the tEMR.	154	**5 (2)**	5.2	12.3	18.2	8.4	33.8	19.5	2.6
A specific person/group is available for assistance with any difficulties related with tEMR use.	154	**4 (2)**	3.9	17.5	10.4	29.9	18.2	17.5	2.6
**Perceived Usefulness (PU)**									
I find the tEMR to be useful for learning.	154	**5 (1)**	1.3	9.7	5.8	7.8	30.5	35.7	9.1
Using the tEMR enhances my learning in classes and laboratories.	154	**5 (2)**	3.2	9.7	3.2	9.1	26.0	40.9	7.8
Using the tEMR better prepares me for IPPEs.	153	**5 (2)**	2.0	9.8	3.9	15.7	28.8	31.4	8.5
Using the tEMR better prepares me for my APPEs/p4 professional year.	154	5 (2)*	1.9	7.1	3.9	21.4	21.4	36.4	7.8
Using the tEMR improves my pharmacy skills.	154	**5 (2)**	1.9	8.4	5.2	13.6	31.8	31.8	7.1
The tEMR has all the functions and capabilities I expect it to have.	154	**5 (2)**	5.8	11.7	15.6	14.9	27.9	22.1	1.9
**Attitude toward Using (ATT)**									
I think it is worthwhile to use the tEMR for my learning activities.	154	**6 (2)**	2.0	8.5	9.2	5.9	23.5	41.2	9.8
I think it is preferable to use the tEMR rather than traditional, paper-based activities	153	**6 (2)**	6.5	7.2	6.5	9.8	19.6	26.8	23.5
In my opinion, it is desirable to use the tEMR for academic purposes.	153	**6 (2)**	2.0	6.5	7.2	10.5	23.5	35.3	15.0
I have a generally favorable attitude toward using the tEMR for my learning activities.	153	5 (3)*	4.6	10.5	13.1	6.5	26.1	32.7	6.5
**Behavioral Intention to Use (BI)**									
If given a choice, I intend to use the tEMR in the future.	154	**5 (3)**	8.4	13.0	10.4	16.2	20.8	27.9	3.2
If given a choice, I intend to use the tEMR as often as possible.	154	**4 (3)**	13.6	14.9	18.2	22.7	16.9	11.7	1.9

Abbreviations: *IQR* Interquartile range

^a^Median (IQR) analysis for Likert scale items included all response ratings with 1 = strongly disagree and 7 = strongly agree; 8 = N/A

* Significant difference in ratings between pharmacy class responses; *p* < 0.05

### tEMR system quality

Of survey respondents, only 29.7% agreed or strongly agreed that they were satisfied with the use of the tEMR system for student learning. Focus group results support these findings. During focus groups, students reported several challenges with the tEMR interface, including lack of organization that made it difficult for them to complete patient cases. They also found conflicting information and were not sure what the most current information was, such as the patient’s age not always matching the documented date of birth. The students noted that the tEMR contained repetitive information and expressed difficulty with navigating the tEMR to find specific items, such as vital signs.

### Perceived self-efficacy

Forty percent of students agreed or strongly agreed that, after using the tEMR, they felt more confident about using a real EMR as a future pharmacist. Focus group results revealed that the tEMR was beneficial because it provided more realistic patient cases compared to traditional paper cases. Students expressed the importance of seeing a complete, electronic patient profile and agreed the tEMR allowed better insight into the complexity of patients.

### Perceived ease of use

A majority of students (56.1%) somewhat disagreed, disagreed, or strongly disagreed that completing tasks within the tEMR required minimal mental effort. Based on survey results, students reported it was generally easy to locate patients within the tEMR, but it was challenging for them to find the patients’ medications. As one example, students were unable to determine if a medication was a home medication or a medication the patient had discontinued several years ago. Another reported barrier from focus group findings suggest that using the tEMR can be a time-consuming process, especially when a student is looking for a specific piece of information. Students also expressed frustration with search functions within the tEMR, since the system did not always pull up the correct information when they searched by an abbreviation or even the full word.

### Facilitating conditions

Students “neither agreed nor disagreed” (29.9%) that a specific person/group was available for assistance with any difficulties related with tEMR use. They specifically stated it was beneficial when an instructor was able to guide the class using the tEMR (e.g., on a projected screen) about a good starting place or acknowledging where specifically they would be able to find pertinent information. Students also highlighted that having training videos as a reference was beneficial.

### Perceived usefulness

Almost half of respondents (48.7%) agreed or strongly agreed that using the tEMR enhances their learning in classes and laboratories. Of those that completed the survey, 44.2% agreed or strongly agreed that using the tEMR better prepares them for APPE rotations (Table 5). During the focus groups, students specifically emphasized that working with the tEMR gives them more preparation and comfort before both IPPE and APPE rotations. They also reported feeling they are able to develop crucial skills, such as collecting and prioritizing information from an EMR, prior to stepping into their final year of pharmacy school. In addition, students noted the value of seeing how other healthcare professions document patient information within the tEMR.

### Attitude toward using

Approximately 50% of students agreed or strongly agreed that it is preferable to use the tEMR rather than traditional, paper-based activities. More than half of students (51.0%) also agreed or strongly agreed it is worthwhile to use the tEMR for learning activities. There was a significant difference between second year pharmacy and third year pharmacy respondents in having a generally favorable attitude toward using the tEMR for learning activities, with third year pharmacy students being more favorable (Table 3). Focus group discussions provided further evidence that the tEMR allows for more interaction and engages students in active learning.

### Behavioral intention to use

About half (51.9%) of students strongly agreed (3.2%), agreed (27.9%), or somewhat agreed (20.8%) that if given a choice, they would use the tEMR in future coursework. Although the survey did not specify what types of activities the students would like to use the tEMR for in the future, students shared during focus groups that it might be beneficial to follow at least one patient over the entire time they are in pharmacy school. Students described it may be helpful to start out with introductory cases where the patient in the tEMR only has one disease state and then build on that to gradually make the patient cases more complex. Students also expressed interest in documenting notes in the tEMR and using the tEMR for antibiotic or TPN dosing.

### Priorities for tEMR improvement

Students expressed several ways to enhance the tEMR for future use. They highlighted the importance of enhancing the training tools, including going through the tEMR with a live instructor several times before being asked to use the tEMR on their own. Students expressed skepticism about the alignment of the tEMR to real EMRs and believe it is important for the tEMR to resemble a real EMR as much as possible to allow for easier adaptation to rotations and future careers. Furthermore, they suggested some specific improvements to the interface of the tEMR system and mentioned incorporating learning functions, such as immediate, electronic feedback to allow them to know what they missed right away. Finally, students highlighted the importance of faculty members communicating with each other about what specific exposure students have had with the tEMR in order to ensure student activities are meaningful and serve a practical purpose, with continuity across the pharmacy curriculum.

## Discussion

Students expressed several benefits of using the tEMR, including EMR preparation for future APPE rotations and better insights into patient complexity. These findings are similar to the results that Frenzel et al. found when asking P3 pharmacy about their experience with a tEMR. In their study, students anonymously completed an 18-item pre-course and 21-item post-course survey after they used a tEMR for disease state management activities in a pharmaceutical care laboratory course. The authors found that there was a statistically significant difference in students believing that the EMR was user friendly and in students preferring to use an EMR versus a paper-based chart [11]. Our findings support their results since we also found pharmacy students prefer using a tEMR over searching for information in a paper chart. Our study adds to previous literature, because it identified several student learning activities that could be valuable in the future (see in *Behavioral Intention to Use*, above). Our results also provide new, detailed insights into student-reported frustrations, including insufficient tEMR training and difficulty in locating patient’s medications within the tEMR. In order to enhance the tEMR for pharmacy learning, students suggested improving tEMR training, starting with introductory tEMR cases, and increasing complexity over the course of the pharmacy curricula.

Some of our survey items and results were comparable to previous studies conducted in the USA [11–16]. Students found it worthwhile to use the tEMR for their learning activities and expressed increased confidence in their abilities to use real EMRs as a future pharmacist. There were several notable differences amongst cohorts, including how often students in each cohort used the tEMR during the academic year. Both the survey and focus groups were conducted in the spring semester. First year pharmacy students used the tEMR more frequently in the spring semester in their courses, whereas second and third year pharmacy students used the tEMR more frequently in the patient skills lab in the fall semester. This explains the finding that first year pharmacy students used the tEMR most often, and second and third year pharmacy students used the tEMR least often. Most first year pharmacy students have little to no experience with EMRs in general and exposing them earlier on could help instill confidence prior to their IPPEs and APPEs. A consistent finding in both the survey and focus groups was more third year pharmacy students felt the tEMR better prepares them for their APPEs/P4 professional year over first year pharmacy students. This is perhaps because the upcoming APPEs are most salient to third year students. It is also likely that first and second year students are unfamiliar with APPEs and aren’t able to correlate the activities in the tEMR to what they will be doing in practice. Third year pharmacy students likely saw a different value of the tEMR, as they had more experiences to draw upon (prior rotations, internships, etc.).

Several tEMR barriers identified by pharmacy students reflect barriers that clinicians encounter when using EMRs in clinical practice, although students’ skeptical remarks about EMR realism indicate they are not always aware of these similarities. For instance, there is continued debate about the right balance between EMR information versus information overload, and whether to restrict the copy and paste function to reduce “note bloat.” [2] Both pharmacy students and a variety of practicing clinicians report that information is difficult to locate within EMRs [34]. Our findings further support this as students reported that using the tEMR was a time-consuming process, especially when students were searching for a specific piece of information. Education regarding EMRs for novice healthcare professionals will continue to be a major focus across disciplines. Pontefract et al. created a National Working Group in the United Kingdom (UK) to integrate electronic patient records (EPRs) into undergraduate education for healthcare students. They identified six key domains of competence and associated learning outcomes to equip healthcare undergraduates for future work in a digital healthcare environment. The agreed domains were reviewed by experts working in medical education or with EPRs and included digital health, accessing data, communication, generating data, multidisciplinary work, and monitoring/auditing [35]. Pharmacy schools will need to continue to integrate EMRs into labs and coursework to provide realistic environments for EMR training. Pharmacy students saw value in seeing how other healthcare professions document patient information within the tEMR. This finding supports the need for expanded interprofessional education in EMR training.

Comparing our findings to the Pharmacy Patient Care Process yielded several insights. Most activities completed by students in the tEMR were part of the initial “Collect” and “Assess” steps of this process, rather than the “Plan,” “Implement,” and “Follow-up.” [8] Future studies should look at integrating tEMRs into these steps of the PPCP in pharmacy curricula. Findings from our survey and focus groups highlighted that students would like to gain experience with these other steps by write their own notes for patient documentation in the tEMR and simulating a handoff or transition of care. Serag-Bolos et al. used an EMR and found that simulations which include transitions of care enhance pharmacy students’ understanding of the important impact that pharmacists have in ensuring continuity of care by collaborating with members of an interdisciplinary team [36]. The tEMR used by students in our study allows for documentation and addition of notes, however instructors either chose not to use that feature during the first year of tEMR implementation or were unaware that this feature was available. Pharmacy students may benefit from documentation-related activities within the tEMR, especially with inputting SOAP notes as this is the typical format for documentation of ambulatory care visits. While pharmacy students did not document in the tEMR system itself, they reported completing this type of documentation outside of the system (i.e. using an electronic template (e.g., Word or paper template, etc.)).

Our study makes important contributions since we used a mixed-methods approach, including qualitative and quantitative techniques to examine tEMR use in pharmacy education. TAM constructs informed both our survey items and focus group questions, which has not been done in previous studies of tEMR use in pharmacy education. Although several schools of pharmacy have examined student perceptions of a tEMR using a survey, our study is one of the first to conduct focus groups with pharmacy students to assess detailed perceptions of a tEMR [11–16]. Focus group results provided further insights than what could be captured by a survey alone. Unlike other studies, we conducted a single study that sampled students from multiple pharmacy years, increasing the generalizability of our findings.

This study was limited to one pharmacy school in the USA, thus, not all of our findings may generalize to other pharmacy programs, although we do not have evidence that our findings are specific to one university. The Regenstrief tEMR system has been adopted by multiple institutions and disciplines, thus the findings could be applied to those programs. The tEMR system also mirrors the same processes for gathering and assessing information for tEMRs and clinical EMRs generally, so our findings are likely generalizable to other systems. In terms of study methods, we adapted a validated survey and added some questions. We examined the face validity of these new questions via piloting with a sample of students, but further validation would be valuable in future research. Data were collected at a single point in time and future research could study tEMR implementation and students’ perspectives longitudinally. One pharmacy school that incorporated a tEMR into four institutional pharmacy practice modules in the USA had a 74% response rate [12]. Our survey response rate was on the lower end and could reduce the potential generalizability of our survey results. The survey was a voluntary survey; thus, we knew when planning the study that the response rate may be low but attempted to address it by using an incentive. In addition, our mixed methods approach adds beyond what could be gleaned from a typical survey. Our use of focus groups helped us understand participants’ perspectives in a way that a survey alone would not and adds strength to our results. We used screening questions to stratify across all possible student attitudes towards the tEMR so our focus group sample was balanced, which supplements survey results and strengthens our overall findings. We collected data during the first year of tEMR implementation, thus, the barriers we identified may be most useful for pharmacy programs across the world that are considering tEMR implementation or in the early stages of incorporating a tEMR into their curricula. Study results were shared with the College and several barriers associated with the tEMR at this institution have been addressed and/or resolved since this research was completed. Our research findings can inform other colleges of pharmacy that are thinking about implementing a tEMR within the curriculum for the first time. In this article, we examined students’ experiences with the tEMR but research on pharmacy instructors’ experiences is also warranted. Future studies could also examine tEMRs longitudinally in pharmacy education, assess the effects on student performance during APPE rotations, and determine whether students are making fewer EMR-related errors when they begin clinical practice.

## Conclusions

Our study is expected to guide future best practices for integrating tEMRs into pharmacy education. Our results captured students’ perspectives from multiple pharmacy years, and we used the established TAM framework to guide our development of survey and focus group questions. Students reported overall satisfaction with the tEMR, highlighting that, compared to paper cases, the tEMR more closely aligns with clinical practice. They also reported that use of the tEMR helped them feel more confident about their ability to use an EMR in clinical practice. Pharmacy schools and educators should direct future efforts toward examining the impact of tEMR technologies on students’ performance in APPEs and clinical practice. Our findings may be particularly helpful for pharmacy programs in the early stages of tEMR implementation or those seeking to expand tEMR implementation across multiple pharmacy years.

## Supplementary information

**Additional file 1.** tEMR Student Survey.

**Additional file 2.** Student Focus Group Guide.

**Additional file 3.** Student Focus Group Themes and Select Student Quotations and Examples.

## Data Availability

The survey dataset analyzed during the current study is available from the corresponding author upon reasonable request. To maintain participant confidentiality, qualitative transcripts are not available, but example quotes can be found in Online Appendix 3.

## Abbreviations

ACPE: Accreditation Council for Pharmacy Education
APPE: Advanced Pharmacy Practice Experiences
CPOE: Computerized Physician Order Entry
EMR: Electronic Medical Record
EPR: Electronic Patient Record
IT: Information Technology
PPCP: Pharmacists’ Patient Care Process
TAM: Technology Acceptance Model
tEMR: Teaching Electronic Medical Record
UK: United Kingdom
USA: United States of America
