# Supplementary material for: Pharmacy students’ perspectives on the initial implementation of a teaching electronic medical record: results from a mixed-methods assessment
Source: BMC Med Educ. 2020 Jun 9;20:187. doi: 10.1186/s12909-020-02091-8 (PMC7285515; doi:10.1186/s12909-020-02091-8)
Supplement: Supplementary file 1 — Additional file 1. tEMR Student Survey. [file 12909_2020_2091_MOESM1_ESM.pdf]

Appendix 1: tEMR Student Survey

Start of Block: Section I: Pharmacy Practice Skills

For survey items 1-36:
* Indicates items originated from the Fathema (2015) survey; modified wording to fit context of tEMR use (16 items)
** Indicates items originated from the Lewis (1995) Post-Study System Usability Questionnaire (PSSUQ); modified wording to fix context of tEMR use (3 items)
+ Indicates newly created items by research team members (17 items)

---

1 What is/was your year in pharmacy school during 2017-2018?+

- ○ P1
- ○ P2
- ○ P3
- ○ Other

Skip To: End of Survey If What is/was your year in pharmacy school during 2017-2018? = Other

2 What training, if any, did you receive for the teaching electronic medical record (tEMR) **(Select ALL that apply)**?+

- ☐ Class instructor(s) provided guidance
- ☐ Completed online quiz on Blackboard
- ☐ Watched online videos
- ☐ tEMR BINGO
- ☐ None
- ☐ Other ____

---

3 The tEMR training I received was effective. +

- ◯ Strongly disagree
- ◯ Disagree
- ◯ Somewhat disagree
- ◯ Neither agree or disagree
- ◯ Somewhat agree
- ◯ Agree
- ◯ Strong agree
- ◯ N/A

---

4 The tEMR training (instructor guidance, online materials, etc.) was available to me at the **TIME** that I needed it. +

- ◯ Strongly disagree
- ◯ Disagree
- ◯ Somewhat disagree
- ◯ Neither agree or disagree
- ◯ Somewhat agree
- ◯ Agree
- ◯ Strong agree
- ◯ N/A

5 What best describes your experience using the tEMR during the 2017-2018 academic year? +

- ◯ I was asked to use the tEMR, but NEVER actually used it
- ◯ I directly viewed/used, but did not directly enter information in the tEMR
- ◯ I directly viewed AND entered information in the tEMR
- ◯ Other (please describe) ____

6 Overall, about how often did you use the tEMR during the 2017-2018 academic year? +

- ○ Daily
- ○ Weekly
- ○ Monthly
- ○ A few times
- ○ None

7 About how many times did you use the tEMR on your own, outside of lab or class expectations? +

____

8 I have used the tEMR for the following MEDICATION-RELATED activities **(Select ALL that apply)**. +

- [ ] View a MEDICATION history
- [ ] Assess medication-related problems (e.g., drug interactions, contraindications, etc.)
- [ ] Review medication allergies
- [ ] Medication reconciliation
- [ ] Assess medication adherence
- [ ] Simulate communicating a patient's medication-related problem(s) with another healthcare professional
- [ ] Assess cost-benefit, formulary, and/or epidemiology principles to medication-related decisions
- [ ] Verify medication orders
- [ ] Other MEDICATION-RELATED activities ______________________________
- [ ] ⊗ I did NOT use the tEMR for any medication-related activities

9 Based on my experience, the tEMR needs to be improved to effectively support the following MEDICATION-RELATED activities: **(Select ALL that apply)** +

- ☐ View a MEDICATION history
- ☐ Assess medication-related problems (e.g., drug interactions, contraindications, etc.)
- ☐ Review medication allergies
- ☐ Medication reconciliation
- ☐ Assess medication adherence
- ☐ Simulate communicating a patient's medication-related problem(s) with another healthcare professional
- ☐ Assess cost-benefit, formulary, and/or epidemiology principles to medication-related decisions
- ☐ Verify medication orders
- ☐ Other MEDICATION-RELATED activities ____
- ☐ ⊗ The tEMR does NOT need to be improved for MEDICATION-RELATED activities

10 I also used the tEMR for these other activities **(Select ALL that apply)**. +

- [ ] View a MEDICAL history (e.g., diagnoses, health problems)
- [ ] Determine a patient's health-related needs
- [ ] Interpret laboratory test results
- [ ] Assess problems NOT related to medications
- [ ] Simulate a handoff or transition of care
- [ ] Assess or document immunizations
- [ ] Write a note for patient documentation
- [ ] Document other patient-specific information (other than a note)
- [ ] Other activities NOT specific to medications

- [ ] ⊗ I did NOT use the tEMR for any other activities

11 Based on my experience, the tEMR needs to be improved to effectively support these other activities: **(Select ALL that apply)** +

- ☐ View a MEDICAL history (e.g., diagnoses, health problems)
- ☐ Determine a patient's health-related needs
- ☐ Interpret laboratory test results
- ☐ Assess problems NOT related to medications
- ☐ Simulate a handoff or transition of care
- ☐ Assess or document immunizations
- ☐ Write a note for patient documentation
- ☐ Document other patient-specific information (other than a note)
- ☐ Other activities NOT specific to medications

- ☐ ⊗The tEMR does NOT need to be improved for any other activities

End of Block: Section I: Pharmacy Practice Skills

Start of Block: Section II: Opinions on tEMR

tEMR system The following questions ask about your level of satisfaction with the tEMR system.

	Strongly disagree (1)	Disagree (2)	Somewhat disagree (3)	Neither agree or disagree (4)	Somewhat agree (5)	Agree (6)	Strongly agree (7)
12. I am satisfied with the use of the tEMR system for student learning.⁺	○	○	○	○	○	○	○
13. I am satisfied with the tEMR system for finding relevant patient information.⁺	○	○	○	○	○	○	○
14. I am satisfied with the quality of the tEMR's CLINICAL CONTENT for my learning.*	○	○	○	○	○	○	○
15. I am satisfied with the tEMR's learning related-functions (e.g., External Resources, Contact Support, tEMR Tutorials).*	○	○	○	○	○	○	○

Self-efficacy The following questions ask about your perceived self-efficacy with the tEMR.

	Strongly disagree (1)	Disagree (2)	Somewhat disagree (3)	Neither agree or disagree (4)	Somewhat agree (5)	Agree (6)	Strongly agree (7)
16. Overall, I feel confident using the tEMR for my pharmacy classes and laboratories.*	○	○	○	○	○	○	○
17. Overall, I feel confident using the tEMR to work on clinical scenarios.*	○	○	○	○	○	○	○
18. After using the tEMR, overall, I feel MORE confident in my ability to use real electronic medical records (EMRs) as a pharmacist.⁺	○	○	○	○	○	○	○

Page Break

Ease of use The following questions ask about the ease of use with the tEMR.

	Strongly disagree (1)	Disagree (2)	Somewhat disagree (3)	Neither agree or disagree (4)	Somewhat agree (5)	Agree (6)	Strongly agree (7)
19. The on-screen instructions provided by the tEMR are clear.**	○	○	○	○	○	○	○
20. Completing tasks with the tEMR requires minimal mental effort.*	○	○	○	○	○	○	○
21. I found it easy to get the tEMR to do what I want it to do.*	○	○	○	○	○	○	○

Page Break

22 When working on activities, I found it easy to LOCATE the following in the tEMR:[+]

	Strongly disagree (1)	Disagree (2)	Somewhat disagree (3)	Neither agree or disagree (4)	Somewhat agree (5)	Agree (6)	Strongly agree (7)
A. Patient(s)	o	o	o	o	o	o	o
B. Patient information	o	o	o	o	o	o	o
C. Pertinent lab values	o	o	o	o	o	o	o
D. Patient medications	o	o	o	o	o	o	o

Page Break

Conditions The following questions ask about facilitating conditions with the tEMR.

	Strongly disagree (1)	Disagree (2)	Somewhat disagree (3)	Neither agree or disagree (4)	Somewhat agree (5)	Agree (6)	Strongly agree (7)
23. It was easy to learn how to use the tEMR.**	o	o	o	o	o	o	o
24. A specific person/group is available for assistance with any difficulties related with tEMR use.*	o	o	o	o	o	o	o

Usefulness The following questions ask about the usefulness of the tEMR.

	Strongly disagree (1)	Disagree (2)	Somewhat disagree (3)	Neither agree or disagree (4)	Somewhat agree (5)	Agree (6)	Strongly agree (7)
25. I find the tEMR to be useful for learning.*	○	○	○	○	○	○	○
26. Using the tEMR enhances my learning in classes and laboratories.*	○	○	○	○	○	○	○
27. Using the tEMR better prepares me for IPPEs.*	○	○	○	○	○	○	○
28. Using the tEMR better prepares me for my APPEs/P4 professional year.*	○	○	○	○	○	○	○
29. Using the tEMR improves my pharmacy skills.⁺	○	○	○	○	○	○	○
30. The tEMR has all the functions and capabilities I expect it to have.**	○	○	○	○	○	○	○

Page Break

Attitude The following questions ask about your attitude toward using the tEMR.

	Strongly disagree (1)	Disagree (2)	Somewhat disagree (3)	Neither agree or disagree (4)	Somewhat agree (5)	Agree (6)	Strongly agree (7)
31. I think it is worthwhile to use the tEMR for my learning activities.*	○	○	○	○	○	○	○
32. I think it is preferable to use the tEMR rather than traditional, paper-based activities (e.g., patient cases)+	○	○	○	○	○	○	○
33. In my opinion, it is desirable to use the tEMR for academic purposes.*	○	○	○	○	○	○	○
34. I have a generally favorable attitude toward using the tEMR for my learning activities.*	○	○	○	○	○	○	○

Intent The following questions ask about your intent to use the tEMR.

	Strongly disagree (1)	Disagree (2)	Somewhat disagree (3)	Neither agree or disagree (4)	Somewhat agree (5)	Agree (6)	Strongly agree (7)
35. If given a choice, I intend to use the tEMR in the future.*	○	○	○	○	○	○	○
36. If given a choice, I intend to use the tEMR as often as possible.*	○	○	○	○	○	○	○

End of Block: Section II: Opinions on tEMR

Start of Block: Section III: Demographics and EMR Experience

37 What is your age in years?

____

38 Gender:

○ Male

○ Female

○ Prefer not to answer

39 What other EMR/EHR(s) have you been exposed to, if any? **(Select ALL that apply)** If none, indicate none below.

- ☐ Epic
- ☐ Cerner
- ☐ MEDITECH
- ☐ CPSI
- ☐ McKesson
- ☐ Other ____
- ☐ None

40 About how much exposure have you had for these EMRs in total (from previous question)?

- ○ Less than 3 months
- ○ 4 to 6 months
- ○ 7 months to 1 year
- ○ More than 1 year but less than 2 years
- ○ 2 years or more
- ○ None

41 What is your intended practice type after graduation?

- ◯ Industry
- ◯ Ambulatory care
- ◯ Hospital-based pharmacy
- ◯ Community pharmacy
- ◯ Independent pharmacy
- ◯ Research
- ◯ Unsure
- ◯ Other ____

---

42 In the text bow below, please add any other feedback that you would like to provide about the tEMR.

____

____

____

____

____

End of Block: Section III: Demographics and EMR Experience
