# Supplementary material for: Pharmacy students’ perspectives on the initial implementation of a teaching electronic medical record: results from a mixed-methods assessment
Source: BMC Med Educ. 2020 Jun 9;20:187. doi: 10.1186/s12909-020-02091-8 (PMC7285515; doi:10.1186/s12909-020-02091-8)
Supplement: Supplementary file 2 — Additional file 2. Student Focus Group Guide. [file 12909_2020_2091_MOESM2_ESM.pdf]

Appendix 2: Student Focus Group Guide

Welcome and thank you for volunteering to take part in this focus group. We are interested in your point of view regarding the teaching electronic medical record for pharmacy education. We realize you are busy and we appreciate your time.

Introduction: This focus group discussion is designed to gather your input on the teaching electronic medical record, which we will refer to as the "tEMR". The focus group discussion will take no more than 2 hours. We will be audio recording today's discussion so that it can be analyzed in-depth after this session. You have the opportunity to refer to computer displays of the tEMR during the focus group. If you choose to demonstrate actions with the tEMR during the focus group session, your computer screen movements will be recorded. Even though this focus group will be recorded, the research team will take precautions to keep your responses confidential. Electronic recordings will be kept securely and transcribed. After the study is complete, the audio recordings will be deleted. The transcribed notes used for analysis will contain no identifiable information that would allow individual subjects to be linked to specific statements. Please answer as truthfully as possible. It is also important that you refrain from discussing the comments of other group members outside the focus group. If there are any questions or discussions that you do not wish to answer or participate in, you do not have to do so; however please attempt to participate in the discussion as much as possible.

Six Ground rules

i. Please say your assigned number prior to speaking each time you share
ii. You may speak in any order, but only one person should speak at a time
iii. Wait until a person has completely finished their comment before jumping in
iv. You do not have to agree with the views of other people in the group, but be respectful when you disagree with their opinions
v. You can give perspectives from your own experience or the experience of other pharmacy students you know, but please do not reveal the name or any identifying characteristics about any other person's perspectives.
vi. There are NO right or wrong answers.

Do you have any questions before we begin?

15 min Warm up: First, I'd like everyone to introduce himself or herself.

I. <u>(15 min) Current use of tEMR (TAM: Actual Use):</u>

1. In what courses have you used the tEMR?
    - course #
    - # students in course
    - Fall or spring?
2. For each course, what were the types of activities that involved the tEMR?

Interviewer: In this next section of questions, we will discuss the positive aspects of the tEMR.

II. (25min) Benefits/perceived ease of use and usefulness (TAM):

1. What training, if any, did you receive for the tEMR?
2. What aspects of the tEMR training were helpful?
3. What are some things that the tEMR has helped you learn to do?
    a. Probe: What skills have you gained from using the tEMR?
4. Based on your experience, what are the benefits of using the tEMR?
5. In what ways, if any, has the tEMR helped you as a student?
6. What aspects, if any, do you find easy to use with the tEMR?
7. What are the advantages of using the tEMR compared to the usual format for courses?
8. Based on your experience, what are the strengths of the tEMR?
9. What tEMR-related activities do you believe have been the most valuable for preparing you for pharmacy practice?
10. What ideas do you have for future use of the tEMR in pharmacy education?
    a. Probe: what specific activities?

Interviewer: In this next section of questions, we will discuss difficulties to using the tEMR.

III. (25min) Barriers/perceived ease of use and usefulness (TAM):

1. Based on your experience, what are some barriers to your use of the tEMR?
2. Based on your experience, what are some weaknesses of the tEMR?
3. In what ways, if any, does the tEMR hinder your ability to learn?
4. What aspects of the tEMR, if any, do you find difficult to use?
    a. Probe: What aspects, if any, prevent you from using the tEMR to the best of your ability?
5. What are the disadvantages of using the tEMR compared to using the usual format for patient cases?
6. What concerns, if any, do you have about using the tEMR for coursework?
7. In what courses, if any, were you asked to use the tEMR, but experienced difficulty or where unable to use it?
    Probe: Why were you unable to use the tEMR?
8. For what activities, if any, were you asked to use the tEMR, but experienced difficulty or were unable to use it?
    Probe: Why were you unable to use the tEMR?
9. If your use of the tEMR was optional for courses, would you still want to use it?
    Probe: Why or why not?
10. Are there any times you used the tEMR on your own time, when it was *not* required for class? Please describe.

IV. (25min) Ideas for tEMR improvement:

1. What aspects of tEMR training for students, if any, could be improved?
2. In what ways, if any, might the tEMR be improved for your learning as a student?
    a. Probe: What other data, features, or functions, if any, would you like the tEMR to provide to aid students? Aid learning for other pharmacy students?
3. What are some EMR-related skills that you expect to need in pharmacy practice, but that have not yet been included in your coursework?
4. What, if anything, about EMR use in pharmacy practice are you curious about that the tEMR might be used to help address?

10min. Two concluding questions:

1. Anything else that you would like to share about the tEMR?
2. Of all the things we've discussed today, what would you say are the most important issues relating to the tEMR?

Conclusion:

Thank you for participating in this focus group! We hope that you have found this discussion interesting. The experiences you shared will be a valuable asset to this research and are expected to inform future use of the tEMR in the College of Pharmacy. If you have any questions or concerns, please contact Dr. Alissa Russ. Her email is provided in consent. Have a good afternoon/evening!
