# Supplementary material for: Pharmacy students’ perspectives on the initial implementation of a teaching electronic medical record: results from a mixed-methods assessment
Source: BMC Med Educ. 2020 Jun 9;20:187. doi: 10.1186/s12909-020-02091-8 (PMC7285515; doi:10.1186/s12909-020-02091-8)
Supplement: Supplementary file 3 — Additional file 3. Student Focus Group Themes and Select Student Quotations and Examples. [file 12909_2020_2091_MOESM3_ESM.pdf]

Appendix 3: Student Focus Group Themes and Select Student Quotations and Examples		
**Name**	**Definition**	**Supportive Results**
**Theme 1: Current tEMR Use Activities**		
**Collect patient information**	Students were asked to collect patient specific information (medication lists, past medical history, etc.)	• "We normally look at the admission note to get general information about the patient. We will also look at labs that patient may have had. They normally give us a certain date to look at and then we will pull up all notes that the patient may have had on that date." *(Third year pharmacy student)*
**Prepare a care plan/SOAP note**	Students were asked to use the gathered information to create a future plan for the patient within the tEMR	• "Last semester when we used the tEMR, I believe we used it to look up a patient case and then after we used our allotted time to work up the patient, we then did a patient case presentation to a preceptor." *(Third year pharmacy student)*<br>• "We have done cases where we had to go look up a patient and then gather their medication list to prepare for lab or do like a care plan." *(Second year pharmacy student)*
**Medication reconciliation**	The tEMR was utilized to complete medication reconciliations	• "Like when medications were discontinued versus if they were getting refilled. So, like trying to decide, like, if they [patients] are still taking certain medications or not." *(First year pharmacy student)*<br>• "It has primarily been lab, pre-lab assignments or asking to be using the tEMR during labs such as in our medication reconciliation labs." *(Second year pharmacy student)*
**Interpret laboratory values**	Students used the tEMR to understand lab value trends	• "We basically used it to look up patient information, the lab values, help figure out like what are trending clearances, what to evaluate, and whatnot…" *(First year pharmacy student)*
**Theme 2: Benefits of tEMR Use**		
**Exposure to tEMR during training**	Training that was done in person was more beneficial to help with navigation of the tEMR, beneficial to have the videos as a tool	• "I agree videos definitely have some use, because you can go back and re-watch them as opposed to just being taught in a lab and just hope you got everything." *(Second year pharmacy student)*<br>• "…I had lab instructors that did know how to use it pretty well, so that was beneficial for me, I think, because after we worked up the patient case, they went through it on how they would work it up and what they would pull up first, specific labs that they would pull up, so that kind of helped me as I moved forward, too." *(Third year pharmacy student)*

**Interactive learning**	tEMR is interactive and keeps everyone involved in an activity	• "It makes more sense than being given a handout and having all the information there and you just kind of figuring out which part it goes into. So, I feel like without having the tEMR, there is not a lot a student could do in terms of learning how to create a document without having like a practice system." *(First year pharmacy student)*<br>• "Compared to like lecturing to see in the tEMR versus sitting in a lecture and listening, I think it is a huge advantage that it is interactive…" *(Second year pharmacy student)*
**More realistic patient cases**	Using the tEMR is more realistic than using paper cases	• "…my understanding is that these are real patients, just with names changed, and I think that is really cool because in class, you know, we get these kind of cookie cutter case study scenarios, but then we see like true complex patients and realize that some medications are used for things that maybe you didn't learn they were used for, or it just gives you a better insight into patient's complexity." *(First year pharmacy student)*<br>• "I think the tEMR kind of brings life to patients rather than when on a piece of paper…. It kind of makes me think it is like a real patient more than just a paper patient." *(Second year pharmacy student)*
**Provides preparation for pharmacy rotations and future career**	Helps students to prepare for IPPEs, APPEs and real-life practice	• "I think it also gives us a little bit of comfort knowing that we worked with an electronic medical record versus like going [into an] IPPE [rotation] and using it for the first time, or maybe even APPEs and not having used one at all beforehand." *(Third year pharmacy student)*
**Aids development of skills to collect and prioritize information**	Allows students to go through the patient profile and understand what information should actually be used and is relevant	• "Just in general, learning to not overlook small details. Like she said, it could be a patient that changed a dose or just stopped taking medication for a little bit and then came back on it. Because it is not as easy just to say, oh, I saw this medication on their list once, it must still be there." *(First year pharmacy student)*<br>• "…I think it has done a really nice job of helping me to like pick out what information is important and what is not…" *(Second year pharmacy student)*
**Gain experience with filtering and manage information**	With tEMR, you can filter information using functions such as chart search, dates, lab values, etc.	• "I think the biggest benefit the tEMR has is that you can narrow down date ranges to search the documents. So, although you get excessive documents, you can at least find a range." *(First year pharmacy student)*<br>• "I think it is easy to find something from a specific date, or if patient had a lot of information from like a long period of time, their whole life, but you can just

		type in the date and all that information pops up that you need for the day." *(Third year pharmacy student)*
**Exposure to documentation of other healthcare professionals**	Students appreciate the notes that other healthcare professionals enter into the tEMR	• "I think it also was good at teaching us like different professionals in healthcare have notes in the tEMR, so we can see what they are writing and how they are looking up the patient, so seeing different perspectives [of notes]." *(Third year pharmacy student)*
**Learning to navigating an EMR**	Ability to actually practice with it and get a feel for how it works	• "I think the tEMR has helped me learn how to like navigate a patient as far as inside, like a medical record, rather than on a piece of paper because what we usually do is just have a piece of paper with the patient, but with the tEMR we get go onto like a data base and kind of look through almost in a more disorganized fashion than on a paper which would be more realistic in real life." *(Second year pharmacy student)*
**Theme 3: Barriers to tEMR Use**		
**Perceived lack of training**	Frustration with understanding how to navigate the tEMR as minimal training was provided	• "Because right now it's just a lot of us, we are just like, we are really frustrated in how it works, but we are not really sure if that is because we weren't trained well or if it's actually a fault in the system." *(First year pharmacy student)*<br>• "…but it comes down to training. You can't use... a tool can be great, but if you don't know how to use it, it's not useful." *(First year pharmacy student)*
**Challenges with the software interface**	Challenges with user-interface design can take away from time spent working on learning activities	• "I don't know what half the tabs do and if I click on them, I get kicked out of the patient's profile and then I don't know what happened." *(Second year pharmacy student)*<br>• "Then another thing I have noticed is like on the main screen of the patient profile, where it lists their medications, you can't tell if they are like home medications or something they were given a long time in the past…" *(Third year pharmacy student)*
**Lack of organization within the tEMR**	Difficulty with finding information or conflicting information causes frustration for completing patient cases	• "I think it can be kind of confusing when you go in to look at the different chart notes. It like orders all of them. I don't know if it is like chronologically, or how it orders them, but you have to like, if you click on multiple ones, you have to really scroll down through multiple notes in order to be able to read them." *(Third year pharmacy student)*

		• "This is very small, not really significant, but... like, you will have their birthday and then their age, but their age does not match their birthday and I think that confused me…" *(Third year pharmacy student)*
**Time consuming to use tEMR**	Compared to a paper case, it can take a significant amount of time to find information needed to complete a case or assignment	• "I think it is just time consuming to sift through everything." *(First year pharmacy student)*<br>• "Like I think what she is trying to say is more like when you are trying to find one thing in the tEMR and you get stuck and you can't find it and that is the one piece of information you need... you might spend half an hour trying to find this one piece of information. You get so frustrated with it that you don't even finish something." *(First year pharmacy student)*
**Takes away time from patient counseling activities**	Students felt that the focus was shifted to the tEMR and took away from patient counseling activities	• "I almost think that we are focusing on it a little too much. I don't think we've hardly counseled at all in lab this semester which like, I feel like that might be more practical as a learning experience to get grade critiqued on that rather than mastering a fake, or kind of fake EMR that." *(Second year pharmacy student)*
**Frustration with high stakes associated with tEMR assignments**	More concern with getting a good grade and all points on an assignment rather than learning how to navigate tEMR or understanding clinical content	• "I will be too afraid that I am going to lose points and be more focused on the actual grade than the actual learning when in a few years there could actually be a patient in front of me." *(Second year pharmacy student)*
**Lack of easily accessible resources for help**	Students felt that there was not a contact person or a group of people they could reach out to if they needed clarification on using the tEMR	• "I don't really have anyone I could reach out to. I just reach out to whomever is in charge of that lab. I probably wouldn't even do that, but I would just try and figure it out on my own. But yes I don't think there is really a specific person I would go to. I don't really have any resources regarding that." *(Second year pharmacy student)*<br>• "And it is also hard that it is an online course, because it is like you can't really... it's hard... I guess it is hard to get help." *(First year pharmacy student)*
**Mismatch between student and faculty expectations**	Expectations of faculty and students were different, causing difficulties in completing activities	• "…it feels kind as if there is a disconnect between what the instructor think that the students know [about the tEMR] and how to navigate the tEMR, as opposed to how the students feel like they are able to navigate it." *(First year pharmacy student)*

**Searching for patient information**	When searching for a specific item, students were unable to use the chart search function to always find what they were looking for	• "I want to say something about Chart Search. With it being a medical record, it is frustrating that if I wanted... we will use "discontinued" for example. If I wanted to look up what medications are discontinued and I wanted to use Chart Search, if I typed... some notes will actually say "discontinued" written out, and some will say like "D/C" for the abbreviation, and it is frustrating that like when I can't find weight or I can't find what was discontinued, I might have to search like weight, oh, there is nothing in her chart that actually they wrote out the word "weight", better type in "WT:" and see if someone wrote it that way in the note, and it is frustrating that it is not like a smart enough system, being a medical record, to not catch common medical abbreviations when you are trying to use like a control find kind of chart search system." *(First year pharmacy student)*
**Inaccurate or repetitive patient information**	Patient information may have been different in various parts of the chart and some information was included multiple times	• "That is the biggest barrier that I see... a lot of the information doesn't match in different places in the tEMR even. Like the admission note will say something completely different to what is in the labs."<br>• "One thing I have learned from this specific TEMR is it has a lot of repetitive information and I don't know if that is really realistic with all of the EMR's…"<br>• "I think one of the main frustrations that I have with the amount of data that we have to go through is just the amount of repetition and realizing I have spent the entire like two minutes re-reading a paragraph that I read three clicks ago looking to see if something had changed when it really hadn't."
**Difficulty with navigation of the tEMR**	Students not aware of all of the functions of the tEMR and unable to easily navigate to find what they were looking for	• "At least for me, at first, I didn't realize that not all of the boxes for different labs were clicked whenever you would search, so I was missing a lot of the vital information until someone finally told me that you had to go in manually and click it, so that took me a while to figure out." *(Third year pharmacy student)*
**Theme 4: Ideas for Future tEMR Use Activities**		
**Differentiate considerations for use**		• "If they were to use it more, I would say one place it could be beneficial would be in lecture like therapeutics lecture if a professor went through a case that was through the TEMR, I feel like they could hop on like interactions more and kind of make the patients wind up in just like a paragraph case."<br>• "I think definitely starting the tEMR use as a P1, like one of the first things you learn how to do in lab is

		super important because then they can use it throughout their whole time in pharmacy school, and kind of build, like instead of working up one disease state in a patient, like workup multiple disease states, or work up multiple days at a hospital stay, and I think just building on that will prepare them to go out into rotations and then practice, just because that is how you do things in the real world.”
**Advancing tools/methods for training students**	Interest in enhancing training to feel more comfortable in navigating the tEMR	• “I would like to see more training in class or in lab for how to use it.” *(First year pharmacy student)*<br>• “I think having... maybe even doing like the scavenger hunt together as a class with a live instructor who is really knowledgeable about it, and then you could get help on the parts you were struggling on. Like, if you can't figure something out, then they can show you how to find something, so you know for future.” *(Third year pharmacy student)*
**Optimizing/specifying use of the tEMR for types of students**	Targeting specific activities for specific pharmacy cohorts	• “Or even just having like introductory cases. I mean, I understand the practicality of having to be able to identify what is relevant and what's not, but at the same point, trying to learn how to navigate the tEMR while also being introduced to absolutely everything can be overwhelming.” *(Second year pharmacy student)*<br>• “It may be helpful if the questions, at least for points that we have on tEMR, to be more specific, being like this patient on this day has a medication interaction problem, like, can you find it? Or something that is more specific…” *(Second year pharmacy student)*
**Increase fidelity to resemble EMRs used in clinical practice**	Resemblance to a real EMR as possible to use skills learned in school in future practice	• “…we should have it, so it is very similar to what we would actually see. Because right now, that tEMR is the only EMR I have seen, and so if I go out in practice and it's completely different, then this has absolutely no point.” *(Second year pharmacy student)*
**Improvements for tEMR interface**	Improve the tEMR interface to know where information is located and what is means	• “I guess not even just necessarily adding data, but maybe reformatting what that quick bar at that opening site utilizes, making sure that the information is relevant…” *(Second year pharmacy student)*<br>• “… I would really like to be able to see if a medication is, like, actually active, like has it been given? Is it just something that was ordered and has never been

		given? Was it discontinued? Was it taken actually like seven years ago and hasn't been touched since?" *(Third year pharmacy student)*
**More immediate feedback**	Gain feedback quickly to recognize how they can improve for the future	• "So, by having like a you know input, oh, it's wrong, I know, okay, I tried this way, now I tried, you know, this next way, hey, cool, it's right... I know where to find that information. So, I think having like multiple attempts at things so you get immediate feedback is better." *(First year pharmacy student)*
**More advanced student learning activities**	A number of activities that are used by a pharmacist could be done with the tEMR (following a patient over time, TPN, pharmacokinetic dosing, etc.)	• "…I think it would be really interested to follow one patient over the course of our time using the tEMR. I think it would make it more personable like we actually know this patient…" *(Second year pharmacy student)*<br>• "I know there is a different software that people use it for, but like TPN dosing... I know like a lot of pharmacists do that, but I don't know how to do that, and like maybe that is something we will go over, but I know that is pretty common in institutions for pharmacists to do the dosing, so I don't know if that is something that could be incorporated, just to teach students how to do it. I don't think it is covered very heavily here." *(Third year pharmacy student)*
**Learning how to document within the tEMR**	Documenting within the actual tEMR to gain practice for documentation of notes	• "Also, just like actually putting our documentation in there as well. That is something I would struggle with doing." *(Third year pharmacy student)*<br>• "…like it would be really cool if the students could put their note in the system, like add it in there." *(Third year pharmacy student)*
**Better coordination amongst faculty members**	Faculty members can better coordinate to understand activities the tEMR has previously been used for	• "I think getting faculty all on the same page, I don't know what their knowledge is about it, but getting them on the same page so they can see how cool and useful it is, and then maybe that would like spark them to like use it in what they are teaching or some of their assignments." *(Third year pharmacy student)*
